# Identification of d-Galactan-III As Part of the Lipopolysaccharide of *Klebsiella pneumoniae* Serotype O1

**DOI:** 10.3389/fmicb.2017.00684

**Published:** 2017-04-25

**Authors:** Katarina Stojkovic, Valéria Szijártó, Marta Kaszowska, Tomasz Niedziela, Katharina Hartl, Gábor Nagy, Jolanta Lukasiewicz

**Affiliations:** ^1^Laboratory of Microbial Immunochemistry and Vaccines, Department of Immunochemistry, Ludwik Hirszfeld Institute of Immunology and Experimental Therapy, Polish Academy of SciencesWroclaw, Poland; ^2^Arsanis Biosciences GmbHVienna, Austria

**Keywords:** *Klebsiella pneumoniae*, lipopolysaccharide, O-antigen, galactan, d-galactan-I, d-galactan-III, serotype O1, HR-MAS NMR

## Abstract

*Klebsiella pneumoniae* is a Gram-negative, ubiquitous bacterium capable of causing severe nosocomial infections in individuals with impaired immune system. Emerging multi-drug resistant strains of this species and particularly carbapenem-resistant strains pose an urgent threat to public health. The lipopolysaccharide (LPS) O-antigen is the main surface antigen. It contributes to the virulence of this species and determines the O-serotype of *K. pneumoniae* isolates. Among the nine main O-serotypes of *K. pneumoniae*, O1-and O2-type pathogens are causative agents of over 50% of all infections. Serotype O1, the most common O-serotype, expresses complex LPS consisting of d-galactan-I (a polymer built of → 3)-β-d-Gal*f*-(1 → 3)-α-d-Gal*p*-(1 → repeating units) capped by d-galactan-II (built of [ → 3)-α-d-Gal*p*-(1 → 3)-β-d-Gal*p*-(1 →] repeating units). Galactan-I is present as the sole polymer in O2 serotype. Recently, in case of serotype O2, conversion of galactan-I to galactan-III (→ 3)-β-d-Gal*f*-(1 → 3)-[α-d-Gal*p*-(1 → 4)]-α-d-Gal*p*-(1 →) was reported. Substitution of → 3)-α-d-Gal*p* by a branching terminal α-d-Gal*p* was dependent on the presence of the *gmlABC* operon and had a major impact on the antigenicity of the galactan polymer. Genetic analysis indicated that 40% of the O1 clinical isolates also carry the *gmlABC* locus; therefore we aimed to characterize the corresponding phenotype of LPS O-antigens. The presence of galactan-III among O1 strains was proven using galactan-III-specific monoclonal antibodies and confirmed by structural analyses performed using sugar and methylation analysis as well as classical and high-resolution magic angle spinning NMR spectroscopy. By using an isogenic mutant pair, we demonstrated that galactan-III expression was dependent on the presence of glycosyltransferases encoded by *gmlABC*, as was shown previously for the O2 serotype. Furthermore, the galactan-II structures in O1*gml*+ strains remained unaffected corroborating no functional interactions between the biosynthesis of galactan-III and galactan-II polymers.

## Introduction

*Klebsiella pneumoniae* is a Gram-negative, ubiquitous bacterium that is a common colonizer of the human gastrointestinal tract, skin, and the upper airways. It can cause severe nosocomial infections, bloodstream infections, pneumonia, meningitis, and sepsis (Podschun and Ullmann, 1998) mainly in individuals with impaired immune system (e.g., neonates, elderly, immunosuppressed patients) (Gupta et al., 2003). *Klebsiella* infections represent a frequent problem in intensive care units that are associated with high mortality rate (Podschun and Ullmann, 1998). A serious threat to global public health is the spread of carbapenem-resistant *K. pneumoniae* and the emergence of resistance to last resort antibiotics (Centers for Disease Control and Prevention, 2013; Lee et al., 2016). High mortality rates among patients with bacteremia caused by carbapenem-resistant *K. pneumoniae* are attributed to the limited availability of effective antibiotics, restricted to only a few drugs, such as colistin, polymyxin B, fosfomycin, tigecycline, and selected aminoglycosides as well as their combinations (Lee et al., 2016; Munoz-Price et al., 2017).

*K. pneumoniae* typically expresses both, lipopolysaccharide (LPS) and capsular polysaccharide (CPS, K-antigen), which contribute to the virulence of this species. LPS is a main surface antigen built of the O-specific polysaccharide (O-PS) containing different numbers of oligosaccharide repeating units (RU), core oligosaccharide and lipid A. O-PS structures define O-serotypes of *Klebsiella* strains. In contrast to most Gram-negative bacteria, variability of *K. pneumoniae* O-antigens is currently limited to 9 major O-serotypes: O1, O2, O2ac, O3, O4, O5, O7, O8, O12 (Hansen et al., 1999) and a few subtypes within these serogroups (Kelly and Whitfield, 1996). However, the occurrence of modified or novel O-antigen structures has been forecasted recently (Follador et al., 2016; Szijarto et al., 2016). Since O-antigens are far less variable than CPS, *Klebsiella* LPS O-antigens have been suggested as potential target antigens for immunotherapy as an alternative to antibiotic treatment (Rukavina et al., 1997; Trautmann et al., 1997, 2004; Hsieh et al., 2014; Follador et al., 2016; Szijarto et al., 2016).

According to published epidemiological data, O1 and O2 serotypes are causative agents of 50–68% of all *Klebsiella* infections (Trautmann et al., 1997, 2004; Hansen et al., 1999; Follador et al., 2016). O1 and O2 strains express LPS containing O-PS built of homopolymers of galactose (galactans, gal). O1 serotype expresses d-galactan-I (gal-I) built of → 3)-β-d-Gal*f*-(1 → 3)-α-d-Gal*p*-(1 → as the RU and capped by a polymer of an antigenically different galactose disaccharide [ → 3)-α-d-Gal*p*-(1 → 3)-β-d-Gal*p*-(1 →] termed as d-galactan-II (gal-II) (Whitfield et al., 1991; Kol et al., 1992). On the other hand, O2 consists of gal-I only (Whitfield et al., 1992). For both serotypes, the gal-I synthesis is encoded by the *his* linked *rfb* (*wb*) operon (Clarke and Whitfield, 1992; Kelly et al., 1993; Kelly and Whitfield, 1996). Additionally, O1 strains carry a genetically unlinked locus (*wbbYZ*) that is responsible for the synthesis of d-gal-II (Hsieh et al., 2014). It was shown previously for the O2 serotype that d-gal-I can be decorated by stoichiometric and non-stoichiometric addition of O-acetyl or terminal d-galactose (Kelly et al., 1995). Recent studies revealed the frequent occurrence of gal-I backbone RU decorated by the terminal α-d-Gal*p* residue, termed as d-galactan-III (gal-III), within the O2 serogroup and the genetic background for this modification has been identified (Szijarto et al., 2016). It was shown that conversion of gal-I to gal-III is encoded by *gmlABC*, which is carried adjacent to the gal-I-encoding *rfb* (*wb*) operon. Moreover, it was demonstrated that ~40% of the O1 clinical isolates carry the *gmlABC* genes (Szijarto et al., 2016) suggesting the expression of gal-III also within the O1 serotype. In this study we intend to validate the predicted gal-I/gal-III conversion in O1 strains using serological methods and structural analysis of O-specific polysaccharides isolated from clinical isolates as well as from isogenic mutants. The presented data provide further insight into structural modifications of *K. pneumoniae* LPS that may influence binding of therapeutic or diagnostic antibodies.

## Materials and methods

### Bacteria and growth conditions

*K. pneumoniae* O1 (Kp4, Kp16, Kp24, Kp69, Kp71, Kp75, Kp76, Kp88, Kp111) and O2 (Kp30) isolates used in this study were obtained from clinical specimens. Prototype strains PCM-27 (O2 *gml*+) and PCM-11 (O3:K11) were purchased from the Polish Collection of Microorganisms (PCM, Hirszfeld Institute of Immunology and Experimental Therapy, Polish Academy of Sciences, Wroclaw, Poland). Bacteria were grown on Trypcase Soy Agar plates (BioMérieux) and Luria-Bertani (LB) broth. For large scale LPS preparation, strains were cultured in LB broth in a 10 L fermenter (37°C, 200 rpm, aeration of 5 L/min), killed with 0.5% phenol for 2 h at 60°C, washed with water and freeze-dried. For molecular cloning and cultivation of trans-complemented strains (Kp4/pKP100 and Kp4/pSU2718), selective medium supplemented with chloramphenicol (20 μg/ml) was used.

### Gene complementation studies

pKP100 carries *gmlABC* cloned into expression vector pSU2718 (Martinez et al., 1988) as described elsewhere (Szijarto et al., 2016). The clinical isolate Kp4 (O1*gml*–) was transformed by electroporation with either the empty cloning vector or with the recombinant plasmid.

### Lipopolysaccharide and O-specific polysaccharide preparation

LPS from *K. pneumoniae* strains Kp4 and Kp24 and recombinant mutants of Kp4 were isolated by the hot phenol/water method and purified by dialysis and ultracentrifugation as described elsewhere (Lukasiewicz et al., 2010) including a glass-wool filtration step before ultracentrifugation (Szijarto et al., 2016). O-PS was isolated as previously described (Szijarto et al., 2014) with slight modification (Szijarto et al., 2016). Briefly, poly- and oligosaccharides released by mild acid hydrolysis were ultracentrifuged to remove remains of capsular polysaccharides (6 h, 105,000 × g, 4°C). The obtained supernatants were freeze-dried and fractionated on Bio-Gel P-10 (200–400 mesh) as previously described (Szijarto et al., 2016). Six fractions were obtained and checked by ^1^H NMR spectroscopy. Fractions 1a–1c were identified as O-PS, fraction 2 as shorter O-PS, fraction 3 as core oligosaccharides, and fraction 4 as degradation products of mild acid hydrolysis of labile regions of core oligosaccharide (Szijarto et al., 2016). LPS of clinical isolates were purified with a commercial kit (LPS extraction kit; Intron).

### Immunoblotting

Immunoblotting was performed as described previously (Szijarto et al., 2014). Briefly, purified LPS was blotted onto polyvinylidene difluoride (PVDF) membranes and subsequently incubated with 0.1 or 1 μg/ml monoclonal antibodies (mAb) in TBS containing 0.1% BSA (HyClone) and 0.05% Tween 20 (Fisher Scientific). Murine mAbs 5C10, 1F11, and 5A4, specific to gal-II, gal-I, and gal-III, respectively, were generated by hybridoma technology as described previously (Szijarto et al., 2016). 5C10 and 5A4 were expressed as mouse-human chimeric mAb, and 1F11 was used as murine mAb. Binding of mAbs was detected by horseradish peroxidase-conjugated goat F(ab)2 anti-mouse or anti-human IgG (Southern Biotech) at 1:40,000 dilution in TBS with 0.1% BSA and 0.05% Tween 20. Membranes were developed with ECL Prime Western blotting reagent (GE Healthcare).

### Compositional analysis

Methylation and sugar analyses of O-PS were performed as described earlier (Szijarto et al., 2014) according to the method described by Ciucanu and Kerek (1984).

### NMR spectroscopy

All NMR spectra were obtained using an Avance III 600 MHz (Bruker BioSpin, Germany) spectrometer equipped with a 5 mm QCI cryoprobe with *z*-gradients (O-PS). NMR spectra of isolated O-PSs were obtained for ^2^H_2_O solutions at 25°C using acetone as an internal reference (δ_H_/δ_C_ 2.225/31.05 ppm), processed and analyzed as described earlier (Szijarto et al., 2014). Briefly, signals were assigned based on 1D ^1^H NMR spectra and 2D experiments COSY, clean-TOCSY, NOESY, HMBC, HSQC-DEPT, and HSQC-TOCSY. The excitation sculpting pulse sequence was used for suppression of water resonances. The mixing times in clean-TOCSY experiments were 30, 60, and 100 ms. The delay time in HMBC was 60 ms, and the mixing time for NOESY was 200 ms. The processed spectra were assigned with the use of SPARKY (Goddard and Kneller, 2001). NMR spectra of LPS (Kp4/pSU2718 mutant) were obtained using ^1^H, ^13^C high-resolution magic angle spinning (HR-MAS) probe with z-gradients at the magic angle. LPS (3–4 mg) was suspended in ^2^H_2_O and placed into the ZrO_2_ rotor. HR-MAS NMR experiments were carried out at a spin rate of 4 kHz at 30°C (the measured temperature of the bearing air used for sample spinning). One-dimensional spectra of LPS were acquired with a Carr-Purcell-Meiboom-Gill, CPMG, 90°–(τ−180°−τ)n-acquisition pulse sequence (total delay time 1.2 ms) as T2-filter to remove the broad signals from lipids (Jachymek et al., 1999).

## Results

### Immunological detection of gal-III subunit in O1 serotype strains

**Figure 1:**
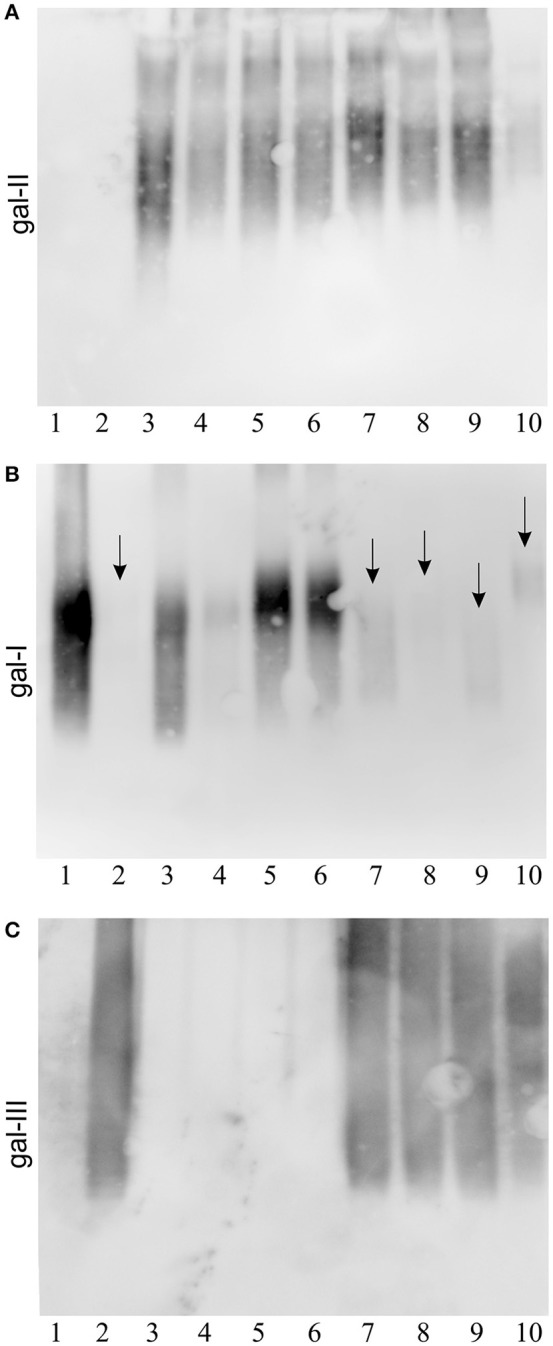
**Immunoblotting of ***K. pneumoniae*** O1 strains with different ***gml*** genotypes**. Separated LPS molecules reacted with monoclonal antibodies specific to different galactan structures as shown: **(A)** anti-gal-II mAb 5C10, **(B)** anti-gal-I mAb 1F11, **(C)** anti-gal-III mAb 5A4. Lanes description: 1, Kp30 (O2*gml–*), 2, PCM-27 (O2*gml*+), 3, Kp16 (O1*gml*–), 4, Kp69 (O1*gml*–), 5, Kp76 (O1*gml*–), 6, Kp111 (O1*gml*–), 7, Kp24 (O1*gml*+), 8, Kp71 (O1*gml*+), 9, Kp75 (O1*gml*+), 10, Kp88 (O1*gml*+). Serotype O2 strains were used as controls (lanes 1 and 2). Arrows indicate the low reactivity of anti-gal-I mAb with the LPS of *gml*+ strains.

LPS was purified from 8 *K. pneumoniae* O1 isolates (4 isolates of respective *gml*+ and *gml–* genotypes) (Figure 1). All strains investigated here were confirmed to belong to O1 serogroup by reactivity with the gal-II specific mAb 5C10 (Figure 1A). Additionally, isolated LPS samples were tested for reactivity with gal-I (mAb 1F11) and gal-III (mAb 5A4) specific mAbs in immunoblots (Figures 1B,C). LPS from *gml–* strains reacted with 1F11 but showed no binding by 5A4 mAb. On the other hand LPS samples from *gml*+ strains were recognized by 5A4 and showed varying degrees of binding to 1F11 (highlighted by arrows in Figure 1B). The retained reactivity to 1F11 in *gml*+ strains confirms non-stoichiometric conversion of gal-I RU to gal-III RU, as proven before in case of O2 strains (Szijarto et al., 2016).

These results showed that presence of *gmlABC* in O1 strains correlated with binding of the gal-III specific mAb 5A4. The binding pattern of gal-II and gal-III specific mAbs to the LPS (ladder-like pattern) suggests a modular arrangement of specific subunits. Since gal-II specific mAbs appear to bind to the higher molecular weight fractions, it is likely that gal-II caps gal-III, analogously to gal-I in case of the classical O1 strains (Whitfield et al., 1991).

### Structural analyses

**Figure 2:**
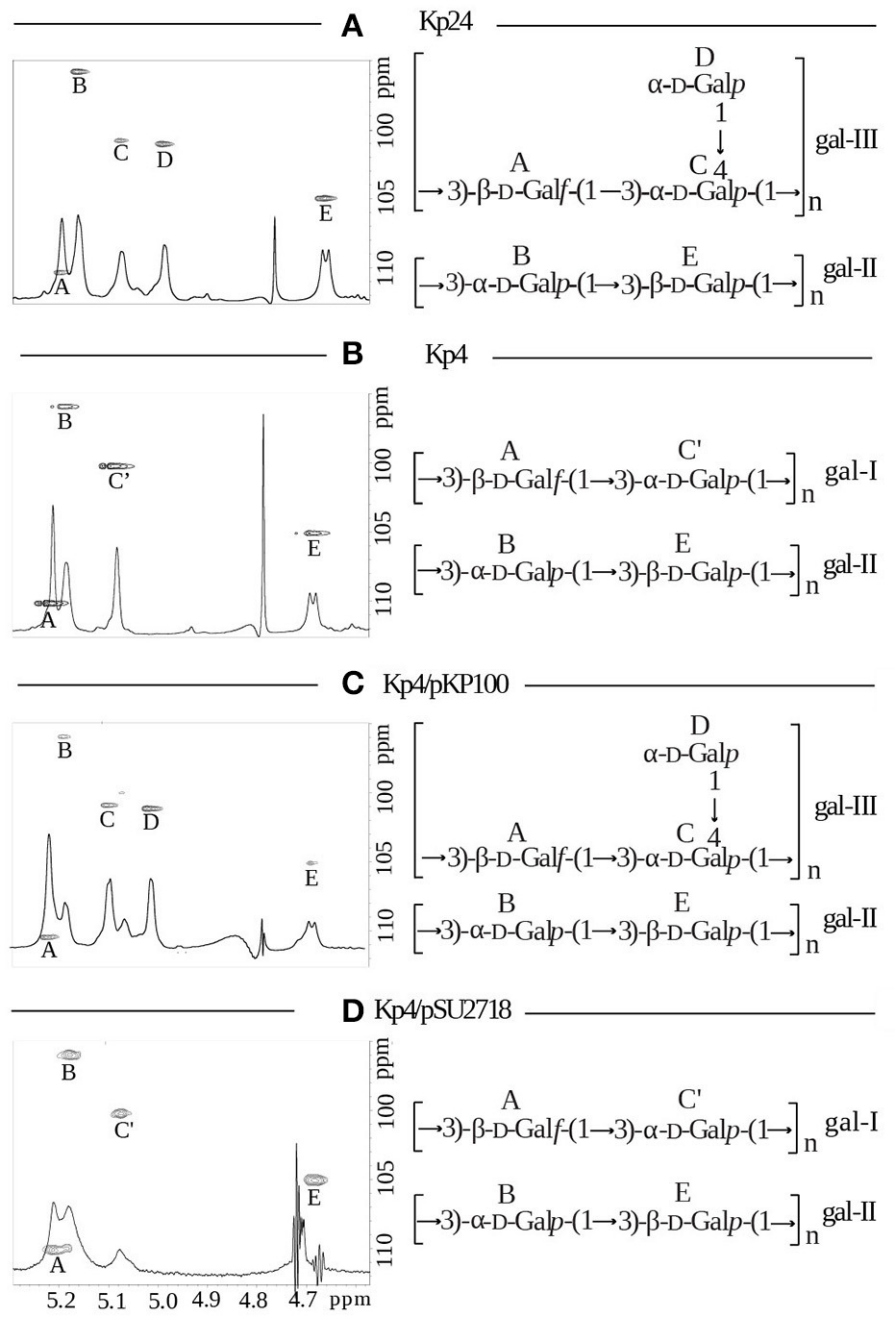
**Structural analysis of lipopolysaccharides from ***K. pneumoniae*** strains: (A)** Kp24 (*gml*+), **(B)** Kp4 (*gml–*), **(C)** Kp4 transformed with pKP100 (recombinant plasmid carrying *gmlABC*), and **(D)** Kp4 transformed with pSU2718 (empty cloning vector). Anomeric regions of ^1^H, ^13^C HSQC NMR spectra overlaid with ^1^H NMR profiles of the isolated O-PS for strains Kp24 **(A)**, Kp4 **(B)**, Kp4/pKP100 **(C)**, and intact LPS for the strain Kp4/pSU2718 **(D)** (left panels). NMR spectra for the strain Kp4/pSU2718 were acquired using HR-MAS technique at a spin rate of 4 kHz at 30°C. The capital letters refer to carbohydrate residues as described in Tables 1, 2. For each strain O-PS gal-I and gal-II repeating units were shown as inset structures (right panels).

The nature of structural modification encoded by *gmlABC* in O1 serotype was determined by NMR spectroscopy, sugar and methylation analyses of the O-PS and LPS isolated from strain Kp24 (O1 *gml*+) (Figure 2A, Table 1) and compared with data obtained for Kp4 (O1 *gml*–) (Figure 2B, Table 2). For the O-PS of the strain Kp24 (O1 *gml*+) sugar and methylation analysis of the polysaccharide showed the presence of 3-substituted Gal*f*, 3-substituted Gal*p*, 3,4-disubstituted Gal*p* and terminal Gal*p* (data not shown). Analyses of the Kp4 (O1 *gml*–) O-PS revealed the presence of 3-substituted Gal*f* and 3-substituted Gal*p*. The complete assignments of ^1^H and ^13^C resonances for both O-PSs were performed by interpretation of one- and two-dimensional NMR experiments (Tables 1, 2), including COSY, TOCSY, NOESY, HMBC, HSQC-DEPT, and HSQC-TOCSY. Chemical shift values for the ^1^H and ^13^C resonances of galactans were compared with data reported previously (Kelly and Whitfield, 1996; Vinogradov et al., 2002; Szijarto et al., 2016).

**Table 1:** **^**1**^H and ^**13**^C NMR chemical shifts and inter-residue connectivities observed for O-PS isolated from ***K. pneumoniae*** LPS from strain Kp24**.

**Residue**	**Description**	**Chemical shifts (ppm)**						**Selected inter-residue NOE and ^3^*J*_*H,C*_ connectivities**	
		**H-1/C-1**	**H-2/C-2**	**H-3/C-3**	**H-4/C-4**	**H-5/C-5**	**H-6a, H-6b/C-6**	**H1/C1 connectivities to**	**Inter-residue atom/residue**
**A**	→ 3)-β-d-Gal*f*-(1 →	5.22	4.34	4.08	4.30	3.86	3.69	3.95	H-3, C-3 of **C**
		110.6	81.4	85.7	80.5	71.0	63.7	77.7	
**B**	→ 3)-α-d-Gal*p*-(1 →	5.19	4.04	4.15	4.29	4.24	3.76	3.80	H-3, C-3 of **E**
		96.1	68.2	80.0	70.0	71.4	61.9	77.7	
**C**	→ 3,4)-α-d-Gal*p*-(1 →	5.10	4.10	3.95	4.18	4.17	3.91	4.08	H-3, C-3 of **A**
		100.9	68.6	77.7	79.1	73.1	61.1	85.7	
**D**	α-d-Gal*p*-(1 →	5.01	3.83	3.92	4.07	4.24	3.79	4.18	H-4, C-4 of **C**
		101.2	69.9	70.0	69.5	71.4	60.9	79.1	
**E**	→ 3)-β-d-Gal*p*-(1 →	4.69	3.75	3.80	4.19	3.68	3.76	4.15	H-3, C-3 of **B**
		105.1	70.4	77.7	65.6	75.7	61.9	80.0	

**Table 2:** **^**1**^H and ^**13**^C NMR chemical shifts and inter-residue connectivities observed for O-PS isolated from ***K. pneumoniae*** LPS from strain Kp4**.

**Residue**	**Description**	**Chemical shifts (ppm)**						**Selected inter-residue NOE and ^3^*J*_*H,C*_ connectivities**	
		**H-1/C-1**	**H-2/C-2**	**H-3/C-3**	**H-4/C-4**	**H-5/C-5**	**H-6a, H-6b/C-6**	**H1/C1 connectivities to**	**Inter-residue atom/residue**
**A**	→ 3)-β-d-Gal*f*-(1 →	5.21	4.42	4.07	4.25	3.87	3.68	3.95	H-3, C-3 of **C'**
		110.2	80.5	85.2	82.6	71.6	63.5	77.8	
**B**	→ 3)-α-d-Gal*p*-(1 →	5.18	4.04	4.15	4.29	4.23	3.73	3.80	H-3, C-3 of **E**
		95.9	68.1	79.9	69.9	71.3	61.7	77.7	
C'^*^	→ 3)-α-d-Gal*p*-(1 →	5.08	3.95	3.95	4.14	4.14	3.73	4.07	H-3, C-3 of **A**
		100.2	68.0	77.8	70.1	72.0	61.7	85.2	
**E**	→ 3)-β-d-Gal*p*-(1 →	4.68	3.74	3.80	4.19	3.68	3.68	4.15	H-3, C-3 of **B**
		105.1	70.5	77.7	65.5	75.6	63.5	79.9	

* *C', represents non-branched variants of residue C (see Table 1)*.

NMR spectra of O-PS of Kp24 (O1 *gml*+) indicated the presence of five anomeric signals corresponding to the residues of gal–III (A, C, D) and gal-II (B, E) polysaccharides.

Residue **A** (δ_H_/δ_C_ 5.22/110.6 ppm, ^1^*J*_C-1,H-1_ 170 Hz) was recognized as the 3-substituted β-d-Gal*f* due to the deshielded furanosic anomeric carbon in β-configuration (δ_C_ 110.6 ppm) and high chemical shift value of C-3 (δ_C_ 85.7 ppm). Residue **C** (δ_H_/δ_C_ 5.10/100.9 ppm, ^1^*J*_C-1,H-1_ 169 Hz) was recognized as the 3,4-disubstituted α-d-Gal*p* due to the large vicinal couplings between H-1, H-2, and H-3 and the small vicinal couplings between H-3, H-4, and H-5 and relatively high chemical shift values of C-3 (δ_*C*_ 77.7 ppm) and C-4 (δ_*C*_ 79.1 ppm). Residue **D** (δ_H_/δ_C_ 5.01/101.2 ppm,^1^*J*_C-1,H-1_ 167 Hz) was recognized as the terminal α-d-Gal*p* due to the large vicinal couplings between H-1, H-2, and H-3 and the small vicinal couplings between H-3, H-4, and H-5.

Residues **B** and **E** were identified as the constituents of gal-II. Residue **B** (δ_H_/δ_C_ 5.19/96.1 ppm, ^1^*J*_C-1,H-1_ 180 Hz) was recognized as the 3-substituted α-d-Gal*p* due to the large vicinal couplings between H-1, H-2, and H-3 and the small vicinal couplings between H-3, H-4, and H-5 and relatively high chemical shift value of C-3 (δ_C_ 80.0 ppm). Residue **E** (δ_H_/δ_C_ 4.69/105.1 ppm, ^1^*J*_C-1,H-1_ 160 Hz) was recognized as the 3-substituted β-d-Gal*p* due to the large vicinal couplings between H-1, H-2, and H-3 and the small vicinal couplings between H-3, H-4, and H-5 and relatively high chemical shift value of C-3 (δ_C_ 77.7 ppm).

The inter-residue connections between the adjacent sugar residues were observed by NOESY and HMBC experiments. The RUs identified for Kp24 O-PS consisted of the → 3)-α-d-Gal*p*-(1 → 3)-β-d-Gal*p*-(1 → disaccharide, reported as gal-II, and the → 3)-β-d-Gal*f*-(1 → 3)-[α-d-Gal*p*-(1 → 4)]-α-d-Gal*p*-(1 → trisaccharide reported as gal-III, which has been published previously as a glycoform of gal-I in O2 serotype (Kelly et al., 1995; Szijarto et al., 2016).

Unlike the O-PS isolated from Kp24, Kp4 (O1 *gml*–) O-PS contained gal-I structure (B and E residues) devoid of a branching terminal α-d-Gal*p*. Interpretation of the complete set of NMR spectra of Kp4 O-PS, including NOESY and HMBC experiments, revealed the presence of C' and A residues attributed to gal-I RU: → 3)-β-d-Gal*f*-(1 → 3)-α-d-Gal*p*-(1 →, as well as E and B residues attributed to gal-II RU: → 3)-α-d-Gal*p*-(1 → 3)-β-d-Gal*p*-(1 → (Table 2, Figure 2B). The RU structures of gal-I and gal-II were in agreement with previously published data (Whitfield et al., 1991; Kol et al., 1992).

### Verification of genetic determinant

**Figure 3:**
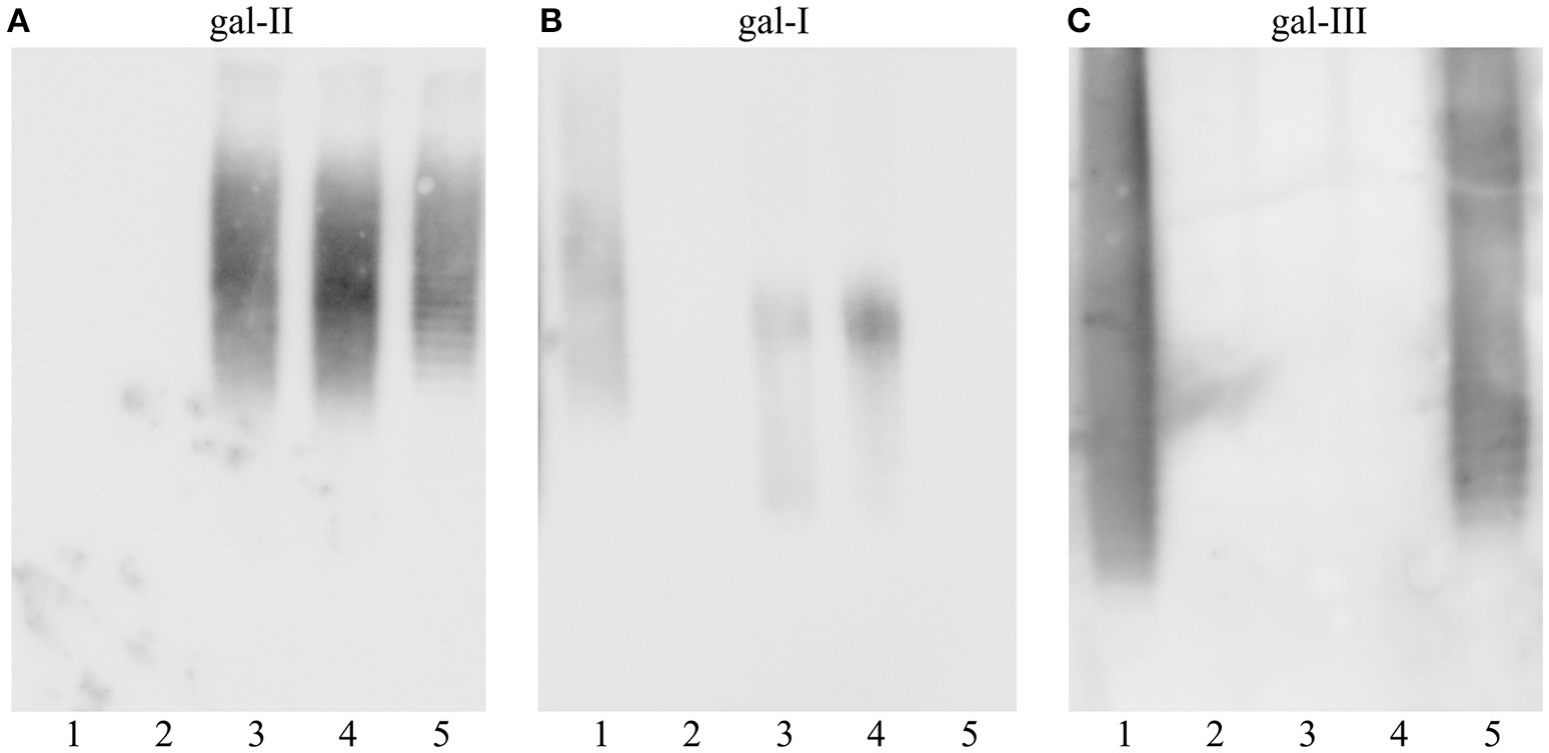
**Immunoblot reactivity of isogenic ***gml*** mutants of ***K. pneumoniae*** O1 strain**. The wild-type (*gml–*) strain Kp4 was trans-complemented with the cloned *gml* operon or the empty vector (as control). Separated LPS molecules were reacted with monoclonal antibodies specific to different galactan structures as shown: **(A)** anti-gal-II mAb 5C10, **(B)** anti-gal-I mAb 1F11, **(C)** anti-gal-III mAb 5A4. Serotype O2 (PCM-27) and O3 (PCM-11) strains were used as controls (lanes 1 and 2). Lane description: 1, PCM-27 (O2*gml*+), 2, PCM-11 (O3, control), 3, Kp4 (O1*gml*–), 4, Kp4/pSU2718 (O1*gml*–), 5, Kp4/pKP100 (O1*gml*+).

To corroborate the exclusive role of *gmlABC* genes in the immunological and structural difference between O1 *gml–* and *gml*+ strains, an isogenic strain pair was investigated. Kp4 (O1 *gml*–) was transformed with pKP100 (carrying *gmlABC*) or with the empty vector (pSU2718). Binding of gal-I, gal-II and gal-III specific mAbs to the LPS of these transformant pairs was tested in immunoblot (Figure 3). While mAb 1F11 recognized LPS from Kp4 carrying pSU2718 (similar to the wild-type strain), no binding was detected to Kp4/pKP100 LPS (Figure 3B). In contrast, 5A4 reacted solely with LPS from Kp4 trans-complemented with *gmlABC* on pKP100 and wild type O2 *gml*+ used as the control (Figure 3C). These data confirm that *gmlABC* genes themselves can modify the gal-I subunits of O1 LPS, similarly to the modification of O2 antigen (i.e., O-PS structure as gal-I without being capped by gal-II). As mAb 5C10 (gal-II specific) reacted with both pKP100 and pSU2718 transformed mutants (Figure 3A), the presence of *gmlABC* shows no effect on the expression of gal-II.

O-PS structures of trans-complemented mutants were verified by NMR spectroscopy. Standard or HR-MAS NMR spectra were obtained for isolated O-PS and/or LPS (Kp4/pSU2718 mutant), respectively. For the O-PS of Kp4/pKP100 (*gml*+) five residues were identified indicating the presence of the disaccharide RU of gal-II, → 3)-α-d-Gal*p*-(1 → 3)-β-d-Gal*p*-(1 →, and the branched trisaccharide RU of gal-III, → 3)-β-d-Gal*f*-(1 → 3)-[α-d-Gal*p*-(1 → 4)]-α-d-Gal*p*-(1 → (Figure 2C). The O-PS of strain Kp4/pSU2718 (empty cloning vector) consisted of gal-I and gal-II (Figure 2D). The structures of O-PSs isolated from Kp4/pKP100 and Kp4/pSU2718 LPS differed due to the presence of α-d-Gal*p* (residue **D**) in gal-I RUs. Structural analyses confirmed that this modification was dependent on the presence of the *gmlABC* operon.

## Discussion

The vast majority of *K. pneumoniae* isolates express LPS O-antigens that are homopolymers of mannose (i.e., mannans; O3 and O5) or homopolymers of galactose (i.e., d-galactans; O1 and O2). Among galactans, expression of the structure known as gal-I is associated with serotype O2 (Whitfield et al., 1992), while the same structure capped with the antigenically different gal-II polymer as the outer O-antigen results in serotype O1 (Whitfield et al., 1991; Kol et al., 1992). Recently, based on the correlation of reactivity patterns of monoclonal antibodies with corresponding genetic background, a locus (*gmlABC*) responsible for the modification of gal-I RU by terminal Gal*p* was described (Szijarto et al., 2016). Structural analysis revealed that the modified structure is identical to that of the O2(2a,2f,2g) subserotype described earlier by the Whitfield group (Kelly et al., 1995). To highlight the structural and antigenic differences, this modified structure has been termed gal-III. Szijarto et al. also showed that expression of gal-III is strongly associated with the KPC-producing epidemic *K. pneumoniae* lineage ST258 (Szijarto et al., 2016). Interestingly, none of the ST258 strains investigated carried the genetic determinants of gal-II synthesis, hence they were considered to belong to the O2 serogroup.

To find out whether gal-III, similarly to gal-I, can be capped by gal-II, strains that carried simultaneously both loci encoding gal-III synthesis (*gmlABC*) as well as that for gal-II synthesis (*wbbYZ*), were selected by PCR. Structural analysis of extracted LPS or isolated O-PS molecules revealed the presence of both gal-II and gal-III (Figure 2), suggesting that expression of these structures is not exclusive. This was confirmed by immunoblots showing reactivity to both gal-II and gal-III specific mAbs. Furthermore, based on the staining pattern in immunoblotting (Figure 1), gal-II appeared to cap gal-III, i.e., a modular arrangement similar to that of gal-I and gal-II (Whitfield et al., 1991) is likely.

Clinical isolates of the O1 and O2 serogroups represent the absolute majority of all *K. pneumoniae* strains (Alberti et al., 1993; Trautmann et al., 1997; Hansen et al., 1999; Hsieh et al., 2014). Detection of O-antigen by specific polyclonal sera has been the gold standard for the typing of *K. pneumoniae* serotypes. Moreover, O-antigens are considered to be attractive targets for active and passive immunization (Rukavina et al., 1997; Trautmann et al., 2004; Szijarto et al., 2016). Thus, it is important to reveal structural modifications of LPS that may influence serotyping and binding of therapeutic monoclonal antibodies. This study corroborated the model for the classification of galactan-type O-antigens proposed previously by Szijarto et al. (2016). The *wb* cluster encoding gal-I synthesis is indispensable for the synthesis of any O1 or O2 antigens. The gal-I to gal-III conversion, i.e., the addition of a terminal α-d-Gal to the gal-I RUs is encoded by the *gmlABC* locus in both O1 and O2 serotypes. Finally, gal-II can cap both gal-I and gal-III, resulting in both cases in O1 serotype, in a *wbbYZ-*dependent manner. Importantly, the functions encoded by *gmlABC* and *wbbYZ* appear to be completely independent from each other.

Interestingly, similarly to O2 strains (Szijarto et al., 2016), serological data (Figure 1B) shown here indicated non-stoichiometric conversion of gal-I RU to gal-III RU for the wild-type O1 *gml*+ strains. This was not supported by NMR data of Kp24 O-PS, since the interpretation was provided for the most intense signals and all attempts to identify and interpret additional signals indicating non-stoichiometric conversion of gal-I RU to gal-III RU failed due to the week intensity. Results of methylation analysis did not provide useful and unequivocal information, since derivatives of → 3)-α-d-Gal*p* might result not only from non-stoichiometric conversion of gal-I RU to gal-III RU, but also from both sugars of gal-II RU. We assume that the expected non-stoichiometric conversion of gal-I RU to gal-III RU in Kp24 O1 *gml*+ strain may be even lower than that for the recently described O2 *gml*+ strain (~6%), what made stoichiometry tracking difficult. On the other hand, overexpression of *gmlABC* (upon complementation) resulted in loss of reactivity with the gal-I specific mAb (Figure 3C). This suggests that transcriptional regulation of *gmlABC* may influence the stoichiometry of gal-I/gal-III ratio. However, since in case of O1 strains both gal-I and gal-III subunits are capped by the serotype determining gal-II polymer, this observation has diagnostic and clinical relevance rather for *K. pneumoniae* O2 strains. Nevertheless, as O1 O-antigen only occurs as combination of gal-II with gal-III or gal-I polymers, the presence of *gmlABC*-encoded modification might be relevant, regarding immunogenicity of future vaccine candidates based on O-PS of *Klebsiella*.

## Author contributions

KS cultured bacteria, isolated and degraded LPS and O-PS, and contributed to their structural analyses. KS and MK prepared bacteria for HR-MAS NMR studies and analyzed NMR data. KS, MK, and TN performed acquisition of HR-MAS NMR data and optimized parameters. JL designed and coordinated bacterial, LPS and O-PS preparations, modifications, and structural studies and contributed to structural analysis. JL prepared biotinylated O-PS for the selection of monoclonal antibodies. VS designed and performed complementation studies, analyzed genetic, and phenotypic data of bacterial strains. KH isolated LPS from and confirmed phenotype of clinical isolates. JL, GN, and VS wrote the manuscript. VS, KH, and GN were involved in the generation, selection, and characterization of monoclonal antibodies used in this paper.

## Funding

This study was financially supported by Eurostars grant E! 7563–KLEBSICURE. The publication supported by Wroclaw Centre of Biotechnology, programme The Leading National Research Centre (KNOW) for years 2014–2018.

### Conflict of interest statement

VS, KH and GN are employees of Arsanis Biosciences GmbH and hold shares in the company. Results of preparation and structural analyses of O-PSs and LPS consist part of Ph.D. thesis of KS. VS, GN, and JL are inventors in pending patent application related to antibodies targeting a galactan-based O-antigen of *K. pneumoniae*. The other authors declare that the research was conducted in the absence of any commercial or financial relationships that could be construed as a potential conflict of interest.

## Abbreviations

COSY: correlation spectroscopy
HMBC: heteronuclear multiple-bond correlation
HR-MAS: high-resolution magic angle spinning
HSQC-DEPT: heteronuclear single-quantum coherence-distortionless enhancement by polarization transfer
LPS: lipopolysaccharide
mAb: monoclonal antibody
NOESY: nuclear Overhauser effect spectroscopy
O-PS: O-specific polysaccharide
PCM: Polish Collection of Microorganisms
PVDF: polyvinylidene difluoride
RU: repeating unit
gal: galactan
TOCSY: total correlation spectroscopy.
